# Mental disorder and first-time marriage formation among non-Western migrant women: A national register study

**DOI:** 10.1016/j.ssmph.2022.101022

**Published:** 2022-01-10

**Authors:** Melanie Straiton, Kamila Angelika Hynek, Anne Reneflot, Lars Johan Hauge

**Affiliations:** a Department of Mental Health and Suicide, Norwegian Institute of Public Health, PO Box 222, Skøyen, 0213, Oslo, Norway

**Keywords:** Migrant women, Mental disorder, Marriage formation, Mental health services, Socioeconomic inequalities, OPHM, Outpatient mental health care services

## Abstract

**Purpose:**

Studies show that there is a mental health selection into marriage among the general population. This study explored the association between mental disorder and marriage formation among non-Western migrant women living in Norway, and whether the association varied with region of origin, income, education and having a dependent child.

**Methods:**

Using linked national register data, we followed 49,329 non-Western never married migrant women aged 18-60 living in Norway between 2006 and 2014. As a proxy for mental disorders, we investigated whether outpatient mental health service use was associated with marital formation using discrete time logistic regression analyses.

**Result:**

Overall, outpatient mental health service use was associated with lower odds of marital formation, even after controlling for sociodemographic factors. Interaction analyses suggested that the relationship was weaker for South Asian women, who had the highest odds of marriage formation, compared with Sub-Saharan African women, who had the lowest. The relationship was also stronger for women with children and women with low incomes.

**Conclusion:**

Mental health selection effects may depend on the universality of marriage. Since marriage may be associated with psychosocial and economic benefits, it is important to identify and treat mental disorder among non-Western migrant women, particularly those with childcare responsibilities and low income.

## Highlights

• Evidence of a mental health selection into marriage among migrant women.
• Mental health service use was used as a proxy for mental disorder.
• The mental health selection effect appears weaker when marriage is more universal.
• Having a child can further reduce marriage formation among women with a disorder.
• Higher income may buffer the negative impact of disorder on marriage formation.

## 1 Introduction

Mental disorders are more prevalent among unmarried compared with married individuals (Williams, Frech, & Carlson, 2010). Although marriage can provide economic and social resources that improve mental health (Braithwaite & Holt-Lunstad, 2017), individuals with mental disorders are also less likely to marry or to stay married (Blekesaune, 2008; Breslau et al., 2011; Hope et al., 1999; Mastekaasa, 1992; Mojtabai et al., 2017) Health selection appears to vary by gender, age and over time (Hope et al., 1999; Liu & Umberson, 2008; Mastekaasa, 2006), pointing to the potential for other sub-group differences. Despite the well-documented differences in marriage patterns across different migrant groups, particularly non-Western migrants (Adserà & Ferrer, 2015; Andersson et al., 2015; Wiik et al., 2018) and their increased risk of mental disorder (Gilliver et al., 2014), there is a lack of longitudinal research on mental health selection among migrants.

Marriage formation patterns among non-Western migrant women are of particular interest, since it is through progress in gender equality, including women's educational and economic advancement, that has led to lower rates of, or delayed, marriage in Europe (Geist, 2017). A similar pattern is now seen in many parts of the world (Ortiz-Ospina & Roser, 2020). Further, women moving from more patriarchal-based societies may experience an increase in social and economic freedom upon migration to a more egalitarian society such as Norway (Tahir, 2020). This may also result in a change in marriage preferences. At the same time, migrant women are at greater risk of social disadvantage; they experience more poverty, weaker attachment to the labour market and a greater childcare burden than both migrant men and non-migrant women (Delara, 2016; Kawar, 2016; Llácer et al., 2007). They also report more mental health problems (Abebe et al., 2014; Blom, 2017; Jarallah & Baxter, 2019). It is therefore of interest to see how mental disorder relates to subsequent marital formation among migrant women. In this study, we focus on women from Eastern European countries not in the European Union (EU), Asia and Africa who are living in Norway.

### 1.1 Marriage formation in context

Marriage rates around the world, particularly in Western countries have declined significantly in recent decades, although there are some exceptions (Jones, 2017; Ortiz-Ospina & Roser, 2020). This has been accompanied by a decline in fertility, a postponement in marriage and the acceptance of alternative forms of marriage such as cohabitation (Lesthaeghe, 2020; Ortiz-Ospina & Roser, 2020). In Norway, 50% of women were estimated to be unmarried at the age of 50 in 2019 compared with 35% in 1999 (Statistics Norway, 2020a). Although the average age at first marriage has increased considerably in the last forty years from 23 to 33 years for women in 2019 (Statistics Norway, 2020a), the median age of first union (including both cohabitation and marriages) has remained fairly stable at around 22 years (Wiik & Dommermuth, 2011). Migrants however, generally have higher rates of marriage and tend to marry earlier than non-migrants in Norway and other Nordic countries (Andersson et al., 2015; Wiik, 2019). These differences may be due to the popularity of cohabitation as a first union among the majority population. More than 90% of majority couples choose cohabitation compared to 40% of migrant couples coming from the same region of origin (Wiik et al., 2018). This preference for direct marriage over cohabitation as a first union is particularly evident for migrants from Eastern Europe, Asia, Middle East/North Africa and Sub-Saharan Africa. This is unsurprising since migrants, to some extent, may take marriage norms, attitudes and traditions with them when they migrate (Andersson et al., 2015). These regions of the world are characterised by traditional family patterns, with high marriage and fertility rates and where women marry at an earlier age (United Nations et al., 2017).

However, marriage formation differences are not only based on cohabitation preferences or marriage norms but can be influenced by migration policy (Adserà & Ferrer, 2015). When migration policy is strict, marriage may be a route to migration, particularly for women (Beck-Gernsheim, 2011). Thus, marriage rates can differ according to reason for migration. Research also shows that early migration is associated with delayed marriage and greater likelihood of entering a mixed marriage (Adserà & Ferrer, 2015; Lindstrom et al., 2020). This is because they may be more likely to take on the marriage norms and values of the majority population than those arriving later.

### 1.2 Mental health and marital status

Although married migrant women in Norway are less likely to use primary mental health care services for mental health problems than their unmarried counterparts (Straiton et al., 2017), unlike in the general population, there are some studies suggesting married migrant women do not always have better mental health. Among Syrian refugees living in Sweden for example, there are no significant mental health differences between the married and unmarried (Tinghög et al., 2017). Another Swedish study also found that younger married migrant women were at increased risk of using psychotropic medicine compared with their single counterparts (Hollander, Bruce, Burström, & Ekblad, 2011). While it may be that marriage is not necessarily be beneficial for all groups in a society, these studies are cross-sectional and did not focus on marriage in particular. The extent to which these mental health differences are pre-existing is therefore unknown. In the general population, some of the apparent beneficial effects of marriage are explained by selection; that individuals with mental disorders are less likely to marry (Breslau et al., 2011; Mojtabai et al., 2017). Selection effects among migrant women appear to remain unexplored.

Further, it is possible that the relationship between mental disorder and subsequent marriage formation may differ for different groups of migrant women. In predominately patriarchal societies such as South Asia, marriage may be important for acceptance and status in the community, particularly for women (Al-Krenawi & Jackson, 2014; Sharma et al., 2013). Thus, there may be more pressure to find and accept a partner regardless of their attractiveness in terms of age or socioeconomic or health status. As a result, the relationship between mental disorder and marital formation could be weaker among groups where marriage is more universal.

There may be other factors that also influence the relationship between mental disorder and marriage formation. In Scandinavia, women with higher education are more likely to marry (Kalmijn, 2013; Krohn, 2012). A woman's income is also positively related to marriage formation, as a higher income makes them a more attractive partner (Geist, 2017). Higher income or education may also help reduce the impact of mental disorder on a variety of outcomes. Indeed, education level is one of the strongest predictors of workforce participation among individuals with mental disorders (Luciano & Meara, 2014). Another study shows that differences in depressive symptoms between the married and unmarried are greatest at the lowest income levels (Carlson & Kail, 2018). Thus, migrant women with higher education or income could fair better in terms of marriage formation when experiencing mental disorder. In other words, high income and education may moderate the relationship between mental disorder and marriage formation among migrant women.

One final aspect of interest is childbearing. In a Scandinavian context, although childbearing is associated with marriage, many cohabit, have children and then marry (Holland, 2013; Lappegård & Noack, 2015). Around 60% of children born in Norway are born to unmarried mothers, majority of whom are cohabiting (Statistics Norway, 2020b). However, childbearing prior to marriage is less common among migrant women (Dribe & Lundh, 2012). Premarital childbearing is heavily stigmatised among many groups of migrant women (Bacchus, 2017; Hawkey et al., 2018) and may lower ones prospects for marriage. Further, while evidence of the effect of motherhood on the mental health of women is inconsistent (Giesselmann et al., 2018; Holton et al., 2010), it is possible that the double stigma of childbearing outside of marriage and of mental disorders could result in greater consequences in terms of marriage formation for migrant mothers. In other words, having a child could moderate the relationship between mental disorder and subsequent marriage formation among migrant women.

### 1.3 Current study

In this study, we aim to determine if there is a mental health selection effect into marriage among migrant women in Norway from regions characterised by traditional family formation patterns (United Nations et al., 2017; Wiik et al., 2021). We focus on migrant women (defined as those born abroad with two foreign born parents) from Eastern-European countries not in the EU, Middle East/North Africa, sub-Saharan Africa, South Asia and East/South East Asia. Collectively we refer to these groups as ‘non-Western migrant women’. With outpatient mental health (OPMH) service use as a proxy for mental disorder, we hypothesise that:

1) Having a mental disorder will reduce the odds of first-time marriage formation among non-Western migrant women, even after adjusting for sociodemographic variables (age, region of origin, having a dependent child(ren), income level, education level, ongoing education and age of and reason for migration).
2) The relationship between mental disorder and first-time marriage formation will be weaker among migrant groups where marriage is more common.
3) The relationship between mental disorder and first-time marriage formation will depend on specific circumstances such as socioeconomic resources (education and income) and having a dependent child(ren).

## 2 Material and methods

### 2.1 Data sources

This study uses data from four national registries, linked at an individual level through a non-identifiable version of a personal number. All registered residents with at least six months of residence are assigned this personal number, in addition to Norwegian born individuals at birth. Demographic information was extracted from the Central Population Registry, available from 1970. This was used to identify all migrant women from non-EU Eastern European countries, the Middle East/North Africa, Sub-Saharan Africa, South Asia and East/South East Asia, their year of birth and civil status. Information on cohabitating was not available in our data. Education level was extracted from the Education Database. Statistics Norway provided information on income. Finally, the National Database for the Reimbursement of Health Expenses, which contains information about patient contacts, was used to extract information on whether or an individual had attended OPMH services during the study period.

### 2.2 Study population

We used a dynamic study design where all non-Western migrant women, aged 18–60 years living in Norway for at least two consecutive years between 2006 and 2014 were potentially included. Information on marital status was extracted for each year of the study. We selected out all never married women at baseline (2006, year of turning 18 or year of migration to Norway, whichever came first). Our never married population also included women who were cohabiting with a partner. Women were followed until they married, or censored at the end of 2014, the year they turned 61, died or emigrated. Because we wanted to exclude women whose migration was dependent on a marriage occurring, we set an additional criterion of being in the dataset for a minimum of three calendar years for newly arrived migrants whose reason for moving to Norway was family. This is because it is possible to come to Norway on a fiancé visa with the intention of marrying within a few months (UDI, n.d.). If one arrives at the end of one year, the marriage could take place early the following year.

### 2.3 Variables

Outcome: Marriage formation, determined by a change in marital status from unmarried to married.

Exposure: At least one contact with outpatient mental health (OPMH) services was used as a proxy for mental disorder. OPMH services are local specialised services where those with acute mental health problems or who need long-term follow-up, without acute need of hospitalisation, can receive help. A referral from a doctor or psychologist is required. The exposure was time varying, available for each year, although once exposed, individuals were coded as always exposed.

Region of origin: We divided migrant women into five regions: Non-EU Eastern Europe, Middle East/North Africa including Turkey, Sub-Saharan Africa, South Asia & East/South East Asia. We excluded women from other regions due to too few individuals to form a cohesive group.

Age at migration: Calculated based on year of migration minus year of birth and grouped into “Minor” (<18 years) and “Adult” (18+ years).

Reason for migration: Divided into three groups; Refugee, Family and Other (including work, study and other/unknown). Reason for migration was not recorded before 1990 and these migrants were classed as other.

Dependent child(ren) (time-varying): (Yes/No). Based on receipt of child benefit, which is automatically paid to mothers whose children are under the age of 18 years and live in the same household (NAV, 2009).

Age group (time-varying): Age, based on year of birth, was grouped into 4 categories: 18–25, 26–35, 36–45 and 46–60 years.

Education (time-varying): Based on highest level of completed education, was divided into: Higher education or less than higher education/unknown.

Low income (time-varying): (Yes/No) Based on personal income of all unmarried women under 60 in Norway, we defined the threshold for low income as lower than 50% of the median income per year.

Ongoing education (time-varying): (Yes/No) Since younger women have not yet had time to finish education or establish themselves in the labour market, we also controlled for current enrolment in education.

### 2.4 Statistical analyses

For the main analyses, we conducted discrete-time logistic regression, controlling for demographic variables to examine the relationship between use of outpatient mental health services and odds of forming a marital union. Since marital changes are only recorded in our data once per calendar year, we lagged the exposure variable by one year (OPMH service use) so we could be sure that the exposure occurred before the marital union. We also lagged dependent child(ren), education and income. First, we ran separate analyses for each variable while controlling for age. Then in the main model, we included all covariates. To investigate if the strength of the relationship between OPMH service use and marriage formation differed for migrants from different regions, for those with dependent children compared to those without and those with different income and education levels, we conducted separate interaction analyses, controlling for all covariates. We also calculated marginal yearly predicted probabilities for marriage formation across the various combination of the predictor variables and plotted the results. This allowed a visual interpretation of the relationships and to see the absolute, rather than relative, probability of marriage formation according the different combinations of predictor variables.

Finally, as a robustness analysis, we repeated all analyses with only migrants who moved as minors, since those who move younger are more likely to take on marriage norms of the majority population (Adserà & Ferrer, 2015; Lindstrom et al., 2020).

## 3 Results

### 3.1 Population sample

Our population sample consisted of 49,329 migrant women with 244,520 person years. Due to lagging several variables by one year, the first year was redundant in the analyses and thus included 195,191 person years. Women were in the study on average 4.95 years (range: 2–9). During the follow-up period, 9836 women (20%) entered a marriage. Table 1 displays the demographics for the overall sample, and the number and percentage of women who married within the different demographic groups. These are displayed for the last year in the study period, lagged for the lagged variables.

**Table 1:** Demographics of total sample and number and percentage of women marrying within each demographic group.^a^.

	N among total sample (n = 49329)	N Marrying (% within each group) (n = 9839)
OPMH service use		
No	46132	9413 (20.40%)
Yes	3197	426 (13.32%)
Age		
18–25 years	18587	3359 (18.07%)
26–35 years	24264	5619 (23.16%)
36–45 years	4503	704 (15.63%)
46–60 years	1975	157 (7.95%)
Region of origin		
Non-EU Eastern Europe	9239	2275 (24.62%)
Middle East/North Africa	6952	1578 (22.70%)
Sub-Saharan Africa	11290	1559 (13.81%)
South Asia	4138	1135 (27.43%)
East/South East Asia	17710	3292 (18.59%)
Child(ren)		
Dependent child(ren)	8469	1436 (16.96%)
No dependent child(ren)	40860	8403 (20.57%)
Education level		
No higher education	35721	5973 (16.72%)
Higher education	13608	3866 (28.41%)
Income		
Mid-high income level	17290	4756 (27.51%)
Lower income level	32039	5083 (15.87%)
Ongoing education		
No	36073	7557 (20.95%)
Yes	13256	2282 (17.21%)
Reason for migration		
Refugee	16471	3154 (19.15%)
Family	10687	2180 (20.40%)
Other	22171	4505 (20.32%)
Age at migration		
Adult	30816	5977 (19.40%)
Minor	18513	3862 (20.86%)

a Age is shown as age in final year of inclusion. OPMH service use, dependent children, education level and income are shown as final lagged year.

Overall, 3197 women had used OPMH services (6%) and a lower proportion of these women married during the study period compared with those who did not (13% vs 20%). Marrying was most common between 26 and 35 years. Marriage formation was most common among South Asians (28%) and least common among Sub-Saharan Africans (14%). We set Sub-Saharan Africans as the reference group so we could explore whether the selection effect was weaker for those with higher marriage rates (hypothesis 2). Marriage was more common among women with higher education (28%) and those with middle/higher income (28%) than those with lower education (17%) and those with low income (16%). Around one in five women married regardless of their reason for, or age at, migration.

### 3.2 Main analysis

Table 2 shows the yearly odds of marriage formation during follow-up, according to the different demographic variables. The first column (adjusted for age group) shows the relationship between marriage formation and each of the covariates while controlling for age group. Women who had used OPMH services had lower yearly odds of marriage formation than women who had not (OR = 0.65). Marriage formation peaked in the 26–35 years group with this group having more than twice the yearly odds of marrying compared to migrant women under 26 years. Women from Sub-Saharan Africa had lower odds of marriage formation than all other groups. South Asians had the highest, with more than twice the yearly odds of marrying compared to Sub-Saharan African women. Women with dependent child(ren) had two thirds the yearly odds of marring than those without dependent child(ren). Higher education and income were associated with higher odds of marrying while migrating as a minor and being a refugee were associated with lower odds.

**Table 2:** Odds ratios (95% confidence intervals) for marriage formation.

	Adjusted for age group	Fully adjusted model Model
OPMH service use	0.65 (0.59–0.71)***	0.77 (0.69–0.85)***
Region of origin		
Sub-Saharan Africa	1.00	1.00
Non-EU Eastern Europe	1.82 (1.70–1.94)***	1.44 (1.35–1.55)***
Middle East/North Africa	1.71 (1.59–1.84)***	1.59 (1.47–1.71)***
South Asia	2.29 (2.11–2.48)***	2.03 (1.87–2.01)***
East/South East Asia	1.60 (1.50–1.70)***	1.34 (1.25–1.44)***
Age		
18–25 years	1.00	1.00
26–35 years	2.16 (2.06–2.25)***	1.32 (1.25–1.39)***
36–45 years	1.13 (1.04–1.23)***	0.66 (0.60–0.72)***
46–60 years	0.55 (0.47–0.64)***	0.28 (0.24–0.33)***
Dependent child(ren)	0.67 (0.63–0.71)***	0.83 (0.78–0.89)***
Higher education	1.72 (1.64–1.80)***	1.45 (1.38–1.52)***
Low income	0.59 (0.57–0.62)***	0.65 (0.62–0.68)***
Ongoing education	0.75 (0.72–0.78)***	0.64 (0.61–0.68)***
Migrated as minor	0.90 (0.86–0.94)***	0.73 (0.69–0.77)***
Reason for migration		
Refugee	1.00	1.00
Family	1.20 (1.13–1.27)***	1.12 (1.06–1.19)***
Other	1.44 (1.37–1.51)***	1.24 (1.17–1.32)***
Wald chi^2^(df)		2953.38 (15)
Prob > chi^2^		0.000
McKelvey & Zavoina's R^2^		10.01%

***p < 0.001.

To see if the association between OPMH service use and marriage formation was robust, we added all covariates to the model. The adjusted odds ratio for OPMH on marriage formation was 0.78. This relationship was significant, showing that OPMH service use is associated with lower odds of marriage formation among migrant women even after accounting for a variety of sociodemographic variables. All covariates were significant predictors of marriage formation.

To see if OPMH service use had the same association with marital formation across region of origin, having dependent children, different income and education levels, we ran analyses with interaction terms and calculated marginal yearly predicted probabilities. We plotted these yearly predicted probabilities expressed as percentages.

**Fig. 1:**
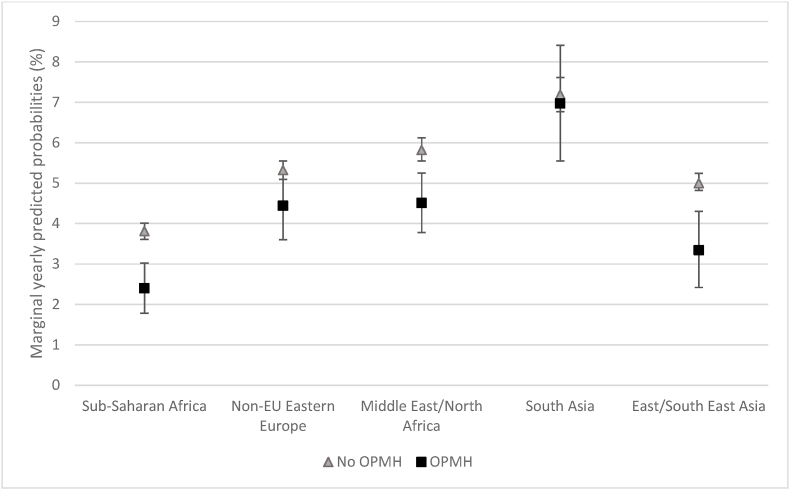
Marginal yearly predicted probabilities (in %) of marriage formation for women with and without OPMH contact by region of origin.

For region of origin, there was a significant interaction between OPMH service use and South Asia (Table 3, model 1). Calculating predicted probabilities confirmed that the association between OPMH service use and marriage formation was far weaker for South Asian women than for the reference group, Sub-Saharan African women. In Fig. 1, we see that for sub-Saharan African migrants, the yearly probability of marriage formation was around 3.8% for those who had not used OPMH services compared with 2.4% for those who had. Thus, those who had not used OPMH services had around 60% higher probability of marrying than women who had. For South Asian women, there was little difference in the yearly probability of marriage formation between those who had used OPMH services and those who had not (7.0% and 7.2% respectively).

**Table 3:** Odds ratio (95% confidence intervals) of marriage formation with interactions^b^.

	Model 1	Model 2	Model 3	Model 4
OPMH service use	0.62 (0.47–0.81)***	0.83 (0.74–0.93)**	0.86 (0.75–0.98)*	0.80 (0.72–0.90)***
Country				
Sub-Saharan Africa	1.00	1.00	1	
Non-EU Eastern Europe	1.43 (1.33–1.53)***	1.44 (1.35–1.55)***	1.45 (1.35–1.55)***	1.44 (1.34–1.55)***
Middle East/North Africa	1.57 (1.46–1.70)***	1.59 (1.47–1.71)***	1.59 (1.47–1.71)***	1.58 (1.47–1.71)***
South Asia	1.98 (1.82–2.16)***	2.03 (1.87–2.21)***	2.03 (1.87–2.21)***	2.23 (1.87–2.21)***
East/South East Asia	1.33 (1.24–1.43)***	1.34 (1.26–1.44)***	1.34 (1.25–1.44)***	1.34 (1.25–1.44)***
Dependent child(ren)	0.84 (0.78–0.89)***	0.85 (0.80–0.91)***	0.83 (0.78–0.89)***	0.83 (0.78–0.89)***
Low income	0.65 (0.62–0.68)***	0.65 (0.62–0.68)***	0.66 (0.63–0.69)***	0.65 (0.62–0.68)***
Higher education	1.45 (1.38–1.52)***	1.45 (1.38–1.52)***	1.45 (1.38–1.52)***	1.46 (1.39–1.53)***
OPMH*Non-EU Eastern Europe	1.33 (0.95–1.86)^a^			
OPMH*Middle East/North Africa	1.23 (0.90–1.70)			
OPMH*South Asia	1.56 (1.09–2.24)*			
OPMH*East/South East Asia	1.06 (0.71–1.58)			
OPMH*Dependent children		0.75 (0.59–0.95)*		
OPMH*Low income			0.80 (0.65–0.97)*	
OPMH*higher education				0.80 (0.63–1.03)^a^
Wald chi^2^(df)	2929.09 (19)	2822.23 (16)	2976.15 (16)	2966.47 (16)
Prob > chi^2^	0.000	0.000	0.000	0.000
McKelvey & Zavoina's R^2^ 10.12%		10.08%	10.10%	9.99%

^a^p<0.1.

*p < 0.05.

**p < 0.01.

***p < 0.001.

b Also adjusted for all covariates (age group, reason for and age at migration and ongoing education).

**Fig. 2:**
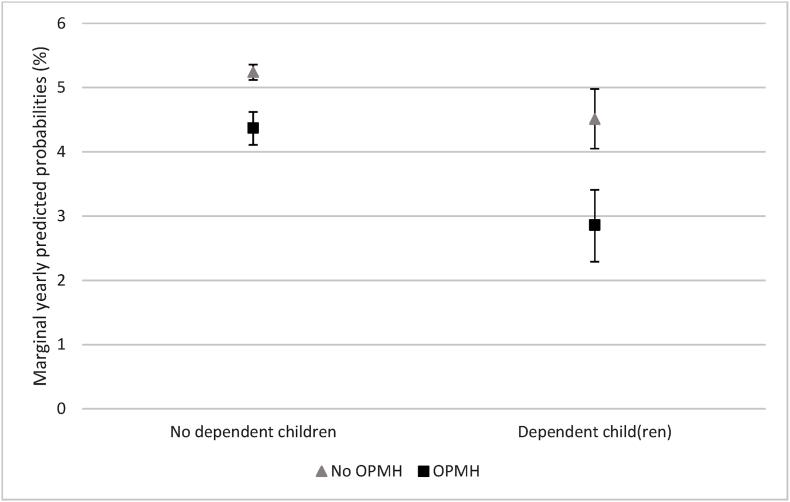
Marginal yearly predicted probabilities (in %) of marriage formation for women with and without dependent children by OPMH contact.

There was also a significant interaction between OPMH service use and having dependent children (Table 3, model 2). Fig. 2 shows the marginal yearly predicted probabilities for women with and without dependent children, and who had or had not used OPMH services. Although OPMH service use was associated with lower probability of marrying for both women with and without children, the difference in probability was greater for women with dependent children (4.5% without OPMH contact and 2.9% with OPMH contract, a 1.6% difference) than for women without dependent children (5.2% and 4.4% without and with OPMH contact respectively, a difference of 0.8%).

**Fig. 3:**
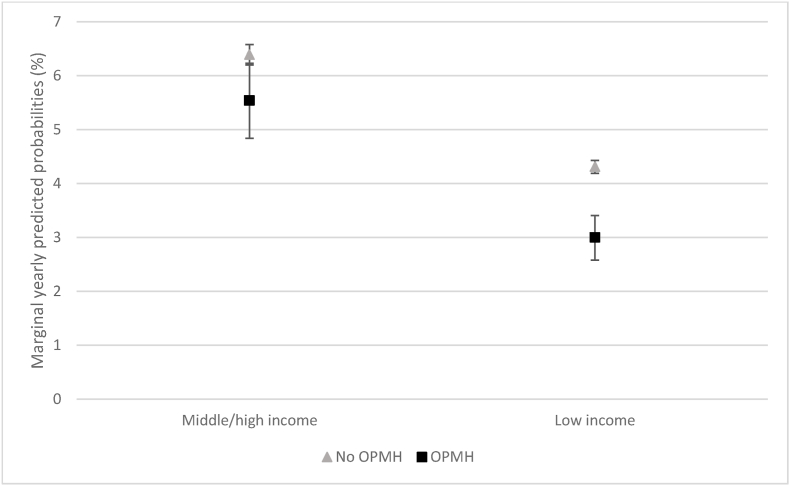
Marginal yearly predicted probabilities in (%) of marriage formation for women by income level and OPMH contact.

For income level, there was also a significant interaction with OPMH on marriage formation (Table 3, model 3). Fig. 3 shows the marginal yearly predicted probabilities for women with low and middle/high income levels by OPMH contact. We see that OPMH service use was associated with a lower yearly probability of marriage formation among those with low income (4.3% for those with no contact and 3.0% with contact). Amongst those with middle or high incomes, the probability of marriage formation was slightly higher for those who did not use OPMH services (6.4%) compared with those who did (5.5%). However, the difference was smaller and the confidence intervals were slightly overlapping indicating that OPMH use had a negative stronger association with marital formation among those with lower income.

**Fig. 4:**
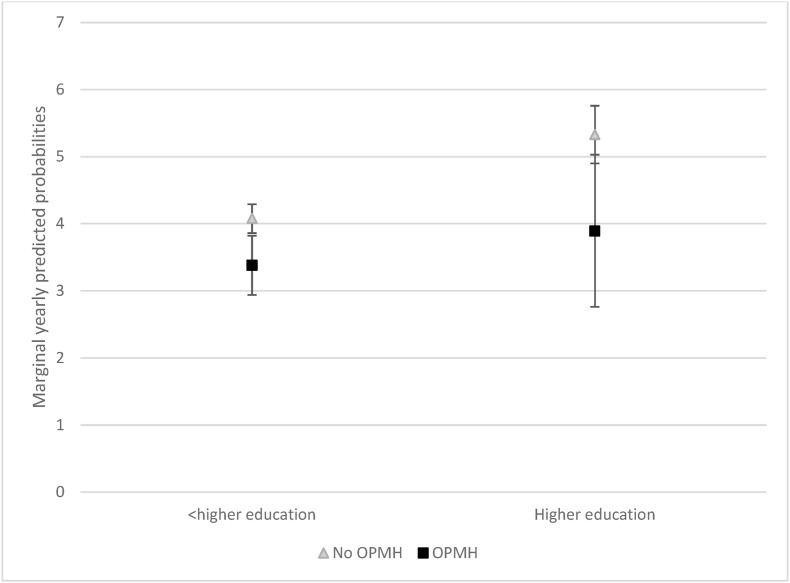
Marginal yearly predicted probabilities in (%) of marriage formation for women by education level and OPMH contact.

Table 3, model 4 suggested there was no significant difference in the association between OPMH and marriage formation by education. However, since the interactions does not tell us anything about the absolute probability, we calculated marginal yearly predicted probabilities for marital formation by education level and OPMH service use. Fig. 4 shows there was no significant difference in marriage formation based on OPMH service use for women with higher education. However, the large confidence interval for women who had used OPMH services may indicate that lack of significant relationship is due to the small number of women with higher education who had used OPMH services. For women without higher education, there was a very slight but significant difference in marriage formation based on OPMH service use; those who had used OPMH services had a 3.4% probability of marriage formation in a given year compared with those 4.1% of those who had not used OPMH services.

### 3.3 Robustness analysis

To evaluate the robustness of our findings, we repeated the analyses with only women who had moved to Norway as minors. See characteristics of the sub-sample in Appendix A. Because 95% of women in this sub-sample were under the age of 36 years, we combined the two oldest age groups in the analyses. Results are shown in Appendix B. Although the odds ratios are not directly comparable with the findings in the main analyses, the pattern of the findings were similar, except that having a dependent child was not associated with marriage formation. The interactions terms between region of origin and OPMH were also in a similar direction to what we found in the main analysis, but none were significant. There was a significant interaction between having a dependent child and OPMH service use on marriage formation. This indicates that while having a child was not associated with marriage formation among women who had not used OPMH services, it was associated with lower odds for women who had. Although income was associated with lower odds of marriage formation, in contrast to the main analysis, there was no significant interaction with OPMH. This may be due to the relative younger age of the women in this sub-sample, many of whom have not had time to establish themselves in the labour market. Again, there was no significant interaction for education and OPMH use on marriage formation indicating that the relationship between OPMH use and marriage formation was similar regardless of education level.

## 4 Discussion

In this study, we investigated whether there is a mental health selection effect into marriage among non-Western migrant women. As hypothesised, we found that women who had attended outpatient mental health care services, a proxy for mental disorder, were less likely to marry than women who had not, even after controlling for a variety of sociodemographic factors (hypothesis 1). We found this association for all migrant women and for migrant women who had moved to Norway as minors, demonstrating the robustness of this finding. Thus, as previously found in studies with the general population (Hope et al., 1999; Mojtabai et al., 2017), mental disorder may impair migrant women's ability to form and maintain a relationship. Since marriage often brings psychological, social and financial benefits (Simon, 2014), it is important to identify and treat mental disorders at an early stage among migrant women, in order to reduce this barrier. This could simultaneously increase social engagement and improve social support for those with a disorder.

Notably, the mental health selection effect appeared significantly weaker for South Asian women compared with women from Sub-Saharan Africa, highlighting sub-group differences. This confirmed our second hypothesis, that the association between mental disorder and OPMH service use would be weaker among groups where marriage is more universal; the probability of marriage was highest among South Asians and lowest among Sub-Saharan Africans. Previous studies on marriage formation also show that South Asians have the highest rates of marriage in Norway (Wiik et al., 2018). Transnational marriages among South Asians resulting in migration are well documented (Charsley et al., 2012). In recent years, the majority of Pakistanis who moved to Norway did so in order to marry a Norwegian resident with Pakistan migrant background (Sandnes & Østby, 2015). However, we purposely attempted to exclude such marriage migrants, who have less opportunity to use mental health services prior to marriage, by only including newly arrived family migrants if they were in the study for more than two years (i.e. did not marry the year of or year after arrival). Thus, high rates of transnational marriages between a new migrant woman and an established Norwegian resident do not explain the lack of association that we found between OPMH service use and marriage formation for South Asians.

While marriage rates have been decreasing around the world (Jones, 2017; Ortiz-Ospina & Roser, 2020) the decline maybe be weaker in South Asia and among South Asians living in other countries (Qureshi et al., 2014). South Asian migrant women tend to marry younger than the majority population and are more likely than other immigrant groups to marry someone with the same background (Dale & Ahmed, 2011; Wiik et al., 2018). Marrying within one's group may somewhat limit the marriage market, which may also encourage a preference for direct marriage rather than other forms of first union, which are more fragile to disruption (Hart et al., 2017). Further, although the practice of arranged marriages may be declining, it is considered more common in South Asia than in other parts of the world (Jones, 2017) and may be practiced among many South Asian migrants living in Europe (Dale & Ahmed, 2011; Pande, 2014)**.** If mental disorder impairs the ability to form and maintain a relationship, then it will have less impact on the likelihood of marriage formation among groups where forming or maintaining a close relationship is not a prerequisite of marriage formation. This may be the case when marriages are arranged, when courtship periods are relatively short or when cohabitation does not precede marriage. Although arranged marriages may also occur in the other migrant groups in this study (Daneshpour, 2016; van Zantvliet et al., 2014), research suggests they are often the exception rather than the norm (Ismail, 2018; Penn, 2011; van Zantvliet et al., 2014). Thus, the greater emphasis on the importance of marriage, coupled with the practice of arranged marriage may explain why OPMH service use was not significantly associated with lower probability of marriage formation among South Asian migrant women.

Our findings also confirm previous research about marriage formation in terms of the importance of socioeconomic resources (Geist, 2017; Kalmijn, 2013). In Norway, women with higher education and higher income are more likely to marry and we found this pattern also holds for non-Western migrant women. Further, we found this both in analyses with all women and analyses with only those moving as minors. This shows the importance of the social context, since research from non-Western countries tends to indicate that women with higher education may be more likely to delay marriage (Jones, 2017; Yaya & Amoateng, 2016). Interestingly, in analyses with all women, we also found that the negative effect of OPMH service use on marital formation was only among those with low income. Thus, higher income may buffer against the negative impact of mental disorder on marriage formation. It may be that income increases the attractiveness of a partner enough to outweigh the negative impact of mental disorder. Alternatively, lower income is associated with greater daily stress (Niemeyer et al., 2019), which can add additional challenges when forming or maintaining a relationship. In analyses with women who moved to Norway as minors, the relationship between OPMH and marriage formation was similar regardless of income level. However, most of the women in this sample were young and therefore many may not yet have been established on the labour market.

Overall, in our study, migrant women with dependent children were less likely to marry than women without children. This is different to what might be expected in the general population. However, in analyses with women moving as minors, there was no association, suggesting marriage formation patterns may be starting to converge with the general population. Migrant women in general who choose to have children outside of marriage may have less traditional values and choose less traditional family formations, such as cohabitation. Thus, this may be why having children is negatively associated with marital formation. Alternatively, migrant women with children may experience stigma attached to having children out of wedlock which could hamper their chances of marriage. Importantly though, we also found that OPMH service use had a stronger association with marriage formation among migrant women with than migrant women without children. This was also the case among women who moved as minors. Thus, childcare responsibilities may increase the burden of coping with a mental disorder and place a greater strain on an existing relationship, or ability to find a potential partner. Research suggests that women with dependent children are more likely to use primary mental health care services than those without dependent children (Stam et al., 2015) and that mothers with a mental disorder are two thirds as likely to marry as mothers without a disorder, even after adjusting for a variety of demographics (Teitler & Reichman, 2008). Migrant mothers may also have limited social and practical support such as help with childcare if their family members live abroad. Thus, unmarried migrant mothers may need extra support not only to prevent detrimental effects on their mental health but also to reduce the negative impact of mental disorder on other life outcomes. Future studies should investigate if sufficient social support and mental health treatment can counteract the disadvantage that migrant mothers with a mental disorder appear to experience.

In this study, we also investigated the role of migration-related factors and found that women migrating as minors were less likely to enter a marriage than women migrating as adults. This could indicate that women moving as children are more likely to adopt union preferences of the majority population, such as cohabitation and is in line with other research (Adserà & Ferrer, 2015; Lindstrom et al., 2020). Refugee women were also less likely to enter a marriage compared with both women moving for family and women moving for other reasons (including work and study). There are two main explanations for this. First, our study shows that mental disorder is associated with decreased odds of marriage formation and other studies show that refugees are at increased risk of mental disorders (Henkelmann et al., 2019). Thus, refugees may be less likely to marry because they are more likely to experience mental disorders. Alternatively, women arriving as refugees, in most cases, get permanent residency, while those arriving for other reasons such as for work or study may only have temporary residency, unless they find a suitable long term job, or a spouse in Norway. Non-refugees may therefore be more motivated to marry than refugees in order to secure residency. Thus, migration policy, to some extent, may influence marriage formation even after excluding marriage migrants.

A major limitation of our study is that we lack information on cohabitation status. Although migrants from the regions we included in this study generally have lower rates of cohabitation than the general population, some do choose to cohabit, especially if their partner is a non-migrant (Wiik et al., 2021). Thus, a proportion of the unmarried women in this study could have been living in a stable cohabiting relationship throughout the whole study period or have formed such a relationship during the study period. It is also possible that some of the women who marry, were previously living with their partner. Cohabitation may be associated with better mental health compared to not living with a partner (Næss et al., 2015). Cohabitors for instance appear to have a lower likelihood of purchasing psychotropic medicine compared with individuals who live alone and, after controlling for age, education, income and children, there are no significant differences between cohabitors and married individuals in purchases of psychotropic medicine (Hedel et al., 2018). Thus, our unmarried group may have a lower rate of mental disorder than what we might expect since some may be living with a partner. This means we may be underestimating the relationship between mental disorder and union formation. Had we been able to identify women who were not in a cohabiting or marital union, the association between mental disorder and subsequent union formation would most likely have been stronger. Thus, our results are not generalisable beyond the association we found between mental disorder and subsequent marriage formation among unmarried migrant women (regardless of partner status). Future research should aim to determine the association between mental disorder and union formation among migrants.

The association may also be somewhat underestimated because our proxy for mental disorder, OPMH service use, only identifies women with the most severe disorders who have sought help and who are not hospitalised. Most common mild to moderate disorders will be treated at the primary care level (Mykletun et al., 2010). Further, barriers to help seeking are well documented among migrants (Debesay et al., 2019; Satinsky et al., 2019; Thomson et al., 2015) and research suggests that migrant women from East/South East Asia and sub-Saharan Africa may have particularly low service use compared to women in the general population (Straiton et al., 2019). It is therefore possible that in these groups, there is a greater proportion of unidentified disorder. Nonetheless, this measure allows national coverage and is not subject to the same selection biases as survey data (Reichel & Morales, 2017). Further, although we excluded all migrant women who were registered as widowed, separated, or divorced upon arrival, we cannot be sure that all marriages are first time marriages. However, this is only likely to be the case if a woman did not correctly report her marital status upon registering her arrival to Norway and is likely to make up a very small proportion of the sample. A final limitation is that due to the nature of the register data, we were unable to identify all factors that could explain the association between mental disorder and marriage formation. It is possible that adjusting for other factors such as family background, partner preferences, religiosity or significant life events could change the association.

## 5 Conclusions

In conclusion, there is support for the mental health selection hypothesis into marriage for non-Western migrant women. However, the strength of the association varies across different groups and may be weaker among groups where marriage is more universal. It is important to identify and treat mental disorders among migrant women, particularly those with childcare responsibilities and low income. These women may experience greater daily stress which can not only impact recovery from a mental disorder but also prevent them from gaining the psychological, social and economic benefits associated with marriage.

## Funding

This study was funded by the 10.13039/501100005416Research Council of Norway (MS: grant number: 273262/H10). The Research Council of Norway had no involvement in the study design, analysis or interpretation of the data. They had no involvement in the writing of this manuscript or decision to submit this article for publication.

## Availability of data and material

The datasets generated and analysed for the current study are not publicly available for data protection reasons. However, the data that support the findings of this study may be available from Statistics Norway and HELFO if ethical approval is granted.

## Ethics approval

Ethical approval for this study was granted by the Regional Committee for Medical and Health Research Ethics, South East Norway (REK 2014/1970) and registry owners approved the use of their data. Consent to participate/publish was not required since this study uses already existing administrative data.

## Declaration of competing interest

On behalf of all authors, the corresponding author states that there is no conflict of interest.
