# Supplementary material for: Mental disorder and first-time marriage formation among non-Western migrant women: A national register study
Source: SSM Popul Health. 2022 Jan 10;17:101022. doi: 10.1016/j.ssmph.2022.101022 (PMC8760389; doi:10.1016/j.ssmph.2022.101022)
Supplement: Multimedia component 1 [file mmc1.docx]

**Appendix A**

| **Demographics of sample of women migrating as minors and number and percentage of women marrying within each demographic group^** | | | |
| --- | --- | --- | --- |
|  | | **N among total sample** | **N** |
|  |  | **(n=18513)** | **Marrying (% within each group) (n=9839)** |
| OPMH service use | |  |  |
| No | | 16376 | 3566 (21.78%) |
| Yes | | 2137 | 296 (13.85%) |
| Age | |  | |
| 18-25 years | | 11138 | 2123 (19.06%) |
| 26-35 years | | 6411 | 1633 (25.47%) |
| 36-45 years | | 864 | 103 (11.92%) |
| 46-60 years | | 100 | 3 (3.00%) |
| Region of origin |  | |  |
| Non-EU Eastern Europe | | 4207 | 1007 (23.94%) |
| Middle East/North Africa | | 4483 | 1024 (22.84%) |
| Sub-Saharan Africa | | 3993 | 456 (11.42%) |
| South Asia | | 2386 | 744 (31.18%) |
| East/South East Asia | | 3444 | 631 (18.32%) |
| Child(ren) | |  |  |
| Dependent child(ren) | | 14995 | 3228 (21.53%) |
| No dependent child(ren) | | 3518 | 834 (18.02%) |
| Education level | |  |  |
| No higher education | | 14291 | 2733 (19.12%) |
| Higher education | | 4222 | 1129 (26.74%) |
| Income | |  |  |
| Mid-high income level | | 8686 | 2580 (29.70%) |
| Lower income level | | 9827 | 1282 (13.05%) |
| Enrolled in education | |  |  |
| No |  | 11058 | 2691 (24.34%) |
| Yes |  | 7455 | 1171 (15.71%) |
| Reason for migration | |  |  |
| Refugee | | 8505 | 1820 (21.40%) |
| Family | | 8633 | 1604 (18.58%) |
| Other | | 1375 | 438 (31.85%) |
| ^Age is shown as age in final year of inclusion. OPMH service use, dependent children, education level and income are shown as final lagged year | | | |
