# Supplementary material for: Mental disorder and first-time marriage formation among non-Western migrant women: A national register study
Source: SSM Popul Health. 2022 Jan 10;17:101022. doi: 10.1016/j.ssmph.2022.101022 (PMC8760389; doi:10.1016/j.ssmph.2022.101022)
Supplement: Supplementary file 2 — Multimedia component 2 [file mmc2.docx]

**Appendix B**

| **Odds ratio (95% confidence intervals) of marriage formation with interactions^1^** | | | | | |
| --- | --- | --- | --- | --- | --- |
|  | **Model 1** | **Model 2** | **Model 3** | **Model 4** | **Model 5** |
| OPMH service use | 0.80 (0.70-0.91)*** | 0.65 (0.43-0.97)*** | 0.89 (0.77-1.02) | 0.79 (0.67-0.93)** | 0.82 (0.71-0.94)** |
| Sub-Saharan Africa | 1.00 | 1.00 | 1.00 | 1.00 | 1.00 |
| Non-EU Eastern Europe | 1.81 (1.60-2.04)*** | 1.78 (1.57-2.02)*** | 1.81 (1.60-2.05)*** | 1.81 (1.60-2.04)*** | 1.81 (1.60-2.04)*** |
| Middle East/North Africa | 1.97 (1.74-2.22)*** | 1.96 (1.73-2.22)*** | 1.97 (1.74-2.23)*** | 1.97 (1.74-2.22)*** | 1.97 (1.74-2.22)*** |
| South Asia | 2.93 (2.56-3.36)*** | 2.86 (2.48-3.29)*** | 2.94 (2.57-3.38)*** | 2.93 (2.56-3.36)*** | 2.93 (2.56-3.36)*** |
| East/South East Asia | 1.20 (1.05-1.36)** | 1.18 (1.03-1.35)* | 1.20 (1.05-1.36)* | 1.20 (1.05-1.36)*** | 1.20 (1.05-1.36)*** |
| Dependent child(ren) | 1.02 (0.93-1.13) | 1.02 (0.93-1.13) | 1.07 (0.97-1.19) | 1.02 (0.93-1.13) | 1.02 (0.93-1.12) |
| Low income | 0.42 (0.39-0.45)*** | 0.42 (0.39-0.45)*** | 0.42 (0.39-0.45)*** | 0.42 (0.39-0.45) | 0.42 (0.39-0.45)*** |
| Higher education | 1.33 (1.22-1.45)*** | 1.33 (1.22-1.45)*** | 1.34 (1.22-1.46)*** | 1.33 (1.22-1.45)*** | 1.34 (1.23-1.47)*** |
| OPMH*Non-EU Eastern Europe | | 1.26 (0.79-2.01) |  |  |  |
| OPMH*Middle East/North Africa | | 1.14 (0.73-1.80) |  |  |  |
| OPMH*South Asia |  | 1.49 (0.90-2.33) |  |  |  |
| OPMH*East/South East Asia | | 1.30 (0.76-2.24) |  |  |  |
| OPMH*Dependent children | |  | 0.65 (0.47-0.88)** |  |  |
| OPMH*Low income |  |  |  | 1.02 (0.79-1.32) |  |
| OPMH*Higher education |  |  |  |  | 0.87 (0.62-1.22) |
| Chi ^2^ (df) | 1008.98 (13) | 1009.74 (17) | 1007.65 (14) | 1009.19 (14) | 1009.66 (14) |
| Prob>Chi^2^ | 0.000 | 0.000 | 0.000 | 0.000 | 0.000 |
| McKelvey & Zavoina's R^2^ | 12.01% | 12.05% | 12.09% | 12.01% | 12.02% |

^1^ Also adjusted for age group, reason for and age at migration and ongoing education, ^p<0.1, *p<0.05, **p<0.01, p<0.001, ***p<0.001
